# Remarks on the Identity of Syphilitic and Gonorrhœal Virus

**Published:** 1809-11

**Authors:** 


### REMARKS *on the* IDENTITY *of* SYPHILITIC *and* GONORRHŒAL VIRUS.

THAT controversy, when properly conducted, is the best means of discovering truth, is I believe generally allowed; it is also, I think, frequently found, that when two opposite opinions are violently contested, truth lies between the extremes. This has, in my opinion, never been more fully exemplified than in the subject of these remarks. This midway of truth, so desirable in every thing, but particularly in medical science, was never more happily hit upon, than in the concluding paragraph of the observations on Mr. Edwards's Case in the Medical and Physical Journal for October, which I shall take the liberty of repeating; and shall also endeavour to draw

 from from it a few axioms, which may *possibly* be nearer the truth, than those of the eminent men who have exercised their pens only on opposite sides of the question. The sentence to which I allude, is the following:

"That there are gonorrhœas from many causes besides venereal poison, some contagious, others not, but that only the venereal gonorrhœa will induce chancre."

From this (and every experiment I have heard of corroborates it) I would deduce the following propositions.

1. That the virus of most gonorrhœas cannot produce syphilis, and cæteris paribus.

2. That the virus of some claps can produce chancre, and ultimately pox.

3. Many of the gonorrhœas, from other causes, are equally contagious with the true venereal gonorrhœa.

4. That syphilitic matter, when applied to the inside of the urethra, produces gonorrhœa.

If these axioms be found true, as they are yet with me mere theory, they will certainly render ours something less of an "ars conjecturalis," than it otherwise would be, and must conduce to an improved treatment of the disorder. All the treatises I have yet read* upon the subject, have been strenuous in the support of one or other of the two opinions; perhaps the practice of them all may be in a great measure right; as the above propositions, if true, do not in any great degree militate against the present treatment. Should any of your numerous correspondents think these cursory remarks worthy of either support or animadversion, their observations will be gladly seen by

JUVENIS.

---

### *To the Editors of the Medical and Physical Journal.*

Gentlemen,

Mrs. J. L. ætat. 26, in October 1805, was seized with violent inflammation and obstruction in the bowels, which was removed by the usual means employed in similar cases; she was naturally of a very costive habit, and after this severe

---

* Had Juvenis favoured us with his name, we could have referred him to a work in which he would find the subject taken up as he wishes.

